# Ameloblastoma Phenotypes Reflected in Distinct Transcriptome Profiles

**DOI:** 10.1038/srep30867

**Published:** 2016-08-05

**Authors:** Shijia Hu, Joel Parker, Kimon Divaris, Ricardo Padilla, Valerie Murrah, John Timothy Wright

**Affiliations:** 1 Pediatric Dentistry, School of Dentistry, University of North Carolina-Chapel Hill, Chapel Hill, NC, USA; 2 Faculty of Dentistry, National University of Singapore, Singapore; 3 Cancer Genetics, University of North Carolina-Chapel Hill, Chapel Hill, NC, USA; 4 Epidemiology, Gillings School of Global Public Health, University of North Carolina-Chapel Hill, Chapel Hill, NC, USA; 5 Diagnostic Sciences, School of Dentistry, University of North Carolina-Chapel Hill, Chapel Hill, NC, USA

## Abstract

Ameloblastoma is a locally invasive benign neoplasm derived from odontogenic epithelium and presents with diverse phenotypes yet to be characterized molecularly. High recurrence rates of 50–80% with conservative treatment in some sub-types warrants radical surgical resections resulting in high morbidity. The objective of the study was to characterize the transcriptome of ameloblastoma and identify relevant genes and molecular pathways using normal odontogenic tissue (human “dentome”) for comparison. Laser capture microdissection was used to obtain neoplastic epithelial tissue from 17 tumors which were examined using the Agilent 44 k whole genome microarray. Ameloblastoma separated into 2 distinct molecular clusters that were associated with pre-secretory ameloblast and odontoblast. Within the pre-secretory cluster, 9/10 of samples were of the follicular type while 6/7 of the samples in the odontoblast cluster were of the plexiform type (*p* < 0.05). Common pathways altered in both clusters included cell-cycle regulation, inflammatory and MAPkinase pathways, specifically known cancer-driving genes such as *TP53* and members of the MAPkinase pathways. The pre-secretory ameloblast cluster exhibited higher activation of inflammatory pathways while the odontoblast cluster showed greater disturbances in transcription regulators. Our results are suggestive of underlying inter-tumor molecular heterogeneity of ameloblastoma sub-types and have implications for the use of tailored treatment.

Ameloblastoma is a slow-growing, locally invasive, benign epithelial odontogenic neoplasm. It is thought to be arise from *SOX2*-expressing dental lamina epithelium^1^, remnants of the tooth-forming enamel organ^2^. The tumor exhibits epithelial cells resembling pre-ameloblasts on a basement membrane in loosely arranged cells resembling stellate reticulum while the stroma consists of loose connective tissue. Although odontogenic tumors are relatively rare, they constitute 3.8% of head and neck pathology, of which 40–50% are ameloblastoma^3^,^4^. Occasionally, ameloblastomas show malignant features or transform into malignancy^5^ and in rare cases metastasize^6^. Current treatment modalities range from conservative enucleation to radical excision and vary according to tumor subtype and location^7^,^8^. High recurrence rates (50–80%) have been observed in cases of conservative treatment^9^. Consequently, and despite recent advances in imaging-assisted surgical margin localization, post-operative histological confirmation is still required. This forces surgeons to either act conservatively, risking the need for a second surgery, or act aggressively thus increasing morbidity^10^ and the need for extensive reconstructive surgery.

There are few genomics and transcriptomics studies of ameloblastoma, with most investigations focusing on candidate-genes. Moreover, different comparison tissues were used in the handful of microarray studies; including gingival tissue^11^,^12^, whole tooth buds^13^, dentigerous cysts^14^ and a universal human reference RNA^15^. Furthermore, whole tumor samples used in these studies included large portions of stromal tissue. In spite of the heterogeneity in comparison tissue, there have been advances in understanding tumorigenesis of ameloblastoma.

A recent study examining the whole transcriptome of ameloblastoma suggested the existence of distinct molecular subtypes^12^. It is envisaged that better understanding of the molecular basis of ameloblastoma can aid the identification of diagnostic and prognostic markers and may lead to the development of novel, personalized treatment protocols^16^. To address this knowledge gap, we embarked on this study aiming to characterize the transcriptome of neoplastic ameloblastoma tissue and identify relevant molecular pathways and genes, using whole genome microarray.

## Methods

### Tumor collection and preparation

The study was conducted in accordance with approved human subject research guidelines and was approved by the local institutional review board and the ethics committee of the University of North Carolina-Chapel Hill. Between 2005 and 2008, 2 fresh frozen samples were obtained during surgical resection and 15 formalin-fixed paraffin-embedded (FFPE) samples were retrieved from the archives of the Department of Oral and Maxillofacial Pathology Laboratory, University of North Carolina (UNC) School of Dentistry. All samples were evaluated by a board-certified oral and maxillofacial pathologist and at least one other author and diagnoses were classified based on the 2005 WHO Histologic Classification of Odontogenic Tumors. Additional demographic data including gender, age, race, and tumor recurrence were recorded and examined for potential associations.

The fresh tumors including the bony resected margins were placed in RNA*later* and Richard Allan Scientifics’ decalcifying solution (water, hydrochloric acid, EDTA, tetrasodium tartrate and potassium tartrate) at 4 °C for 1–4 weeks. They were then frozen at −80 °C before 7 μm sections were obtained under RNAse-free conditions^15^, stained lightly with hematoxylin & eosin and sent for immediate laser capture microdissection (LCM). The FFPE blocks were decalcified before embedding in paraffin. The samples were sectioned, stained and sent for laser capture.

### Laser capture microdissection

The AutoPix^TM^ automated LCM system (Arcturus Engineering, Santa Clara, CA, USA) was used to isolate tumor cells (basal epithelial cells adjacent to the basement membrane). Images of the tissue sections including the captured regions were obtained before and after LCM.

### RNA extraction and microarray

Laser-captured cells from each tumor were pooled and total RNA was isolated with the PicoPure RNA Isolation kit (Arcturus Bioscience, Santa Clara, CA, USA). The Agilent Bioanalyzer 2100 (Agilent Technologies, Palo Alto, CA, USA) was used to assess the yield and quality of total RNA. Amplification was completed on all samples using TargetAmp^TM^ 2-Round Aminoallyl-aRNA Amplification Kit (Epicentre Biotechnologies, Madison, WI, USA).

RNA was then analyzed using the Agilent Whole Human Genome Microarray 4 × 44 K G4112F (Agilent Technologies, Palo Alto, CA) containing 44 thousand 60-mer oligonucleotides representing over 41 thousand human gene transcripts. For this step, 200 ng of RNA was converted into labeled cRNA with nucleotides coupled to fluorescent dye Cy3 using the Low RNA Input Linear Amplification Kit (Agilent Technologies, Palo Alto, CA) according to the manufacturer’s protocol. The Human Universal Reference RNA from Stratagene (Santa Clara, CA, USA) was coupled with Cy5. Cy3-labeled cRNA (1.65 ng) from each sample and the Cy5-labeled universal reference was hybridized to the Agilent whole genome array 41 k formatted chips. Data were extracted using Feature Extraction version 9.5 (Agilent Technologies, Palo Alto, CA). Background subtraction and Loess normalization were performed using default setting of the Agilent extractor. The use of the universal RNA facilitated the use of the dentome as a comparison. It acts as a technical intra/inter normalizing control, decreasing variability by measuring signal output ratio of experimental to reference RNA rather than relying on absolute signal intensity two-color hybridization experiments^17^. The dentome consists of odontogenic tissue (microdissected samples of human odontoblasts, pre-secretory ameloblasts and secretory ameloblasts) expression data from previous work that employed the universal reference as a normalizing control^18^. The data set included 4 samples of each type of odontogenic tissue. It was collected from 12 anterior tooth buds (incisor and canine) from 4 different fetuses with each fetus contributing 3 tooth buds. Each type of odontogenic tissue was collected from a single tooth bud with each of the 3 buds providing a single type of odontogenic tissue or developmental stage. (Gene Expression Omnibus microarray database accession number GSE63289). The ameloblastoma expression data were submitted to the Gene Expression Omnibus microarray database (accession number GSE68531).

### Microarray data analysis

A multiclass analysis was conducted between the 3 types of odontogenic tissue and the 60 genes differentially expressed at a false discovery rate (FDR) <20% were designated as the odontogenic tissue-defining genes. The most appropriate comparison tissue was decided to be the normal tissue with the most similar profile to ameloblastoma, such that identified differences would be tumor specific. Cluster analysis was conducted using Cluster 3.0 between the 3 normal and tumor samples and visualized using Java TreeView-1.1.6r2.

Differential gene expression between tumors and comparison tissue were examined using Significance analysis of microarrays (SAM) 4.0. Ingenuity pathway analysis was used to identify differentially expressed pathways. In addition, upstream analysis from the ingenuity pathway analysis software was conducted. Gene set enrichment analysis (GSEA)^19^ was conducted using GSEA v2.1.0 from the Broad institute (Cambridge, MA, USA) and the “all curated gene sets v4.0” available via the Molecular Signatures Database (MSigDB)^20^. Additionally, the ameloblastoma transcriptome was compared with the 13 cancer molecular subtypes from The Cancer Genome Atlas (TCGA) project^21^ to investigate possible correlation with other known cancer types.

### Microarray gene expression validation using NanoString

A variety of approaches have been used to validate microarray data in the literature and NanoString was selected for the study. A random subset of 3 ameloblastoma and 2 control odontogenic tissue samples was used to validate the microarray gene expression data. NanoString nCounter (Seattle, WA, USA) high throughput gene expression analysis^22^ was performed using the Human Cancer Reference codeset (http://www.nanostring.com/products/gene_expression_panels). Each reaction contained 50 ng of total sample RNA plus reporter and capture probes. Digital counts were extracted, normalized and analyzed using nSolver v2.5 software. Differential expression between ameloblastoma and pre-secretory ameloblast from nanoString was compared with that obtained from the microarray.

## Results

**Figure 1:**
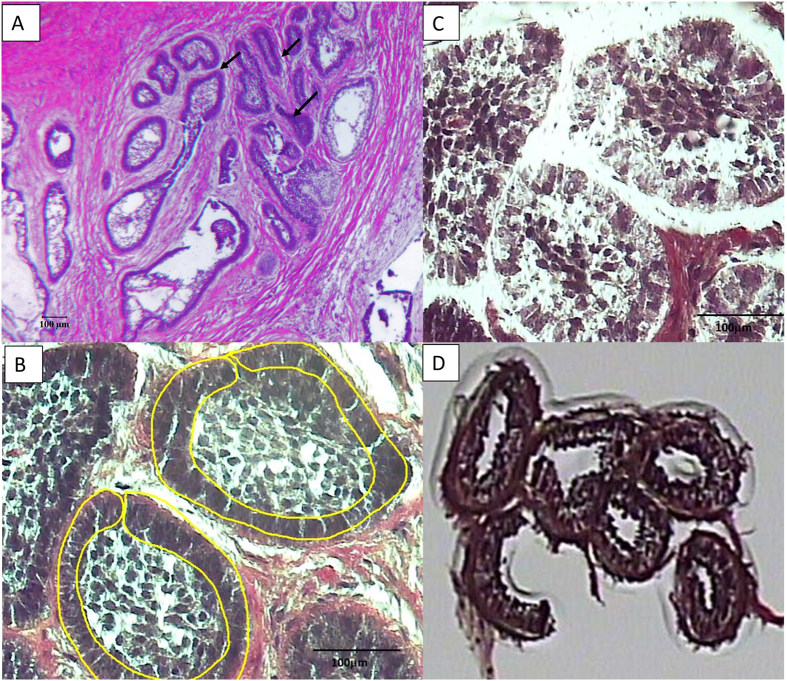
The micrographs show the laser capture of the epithelial portion of an ameloblastoma sample. (**A**) – light micrograph of follicular ameloblastoma at 4X showing tumor epithelial follicles that are single-cell thick with surrounding stroma. Arrows points to tumor follicles, (**B**) – light micrograph of follicular ameloblastoma at 20X showing the laser capture outline of epithelial cells, (**C**) – remnants of the stroma tissue after LCM and (**D**) – captured cells on capsure cap. Scale bar: 100 μm.

LCM facilitated the isolation of basal epithelial (neoplastic) cells from the tumor samples (Fig. 1) without contamination from surrounding stroma cells. RNA was extracted from the LCM samples, with the 260/280 ratio for the 17 samples between 1.7–2.5 and a yield of between 88ng to 928ng (Supplemental Table 1). Quality control analysis conducted on the microarray chips indicated expected values for positive and negative controls, as well as uniformly high detected genes in both the red and green channels. Overall, no outliers were detected in either the normal tissue or tumor arrays indicating consistency in hybridization between samples.

### NanoString validation

Due to the limited RNA quantity available, we did not conduct qPCR validation; instead, the NanoString platform was used to validate microarray gene expression data. Fold changes obtained with the nCounter system were correlated with those obtained from the microarray for the same samples (Supplementary Table 2). The 2 sets of expression data showed a good Pearson correlation (r = 0.61) in the scatter-plot (Supplementary Fig. 1).

### Determination of comparison tissue

**Figure 2:**
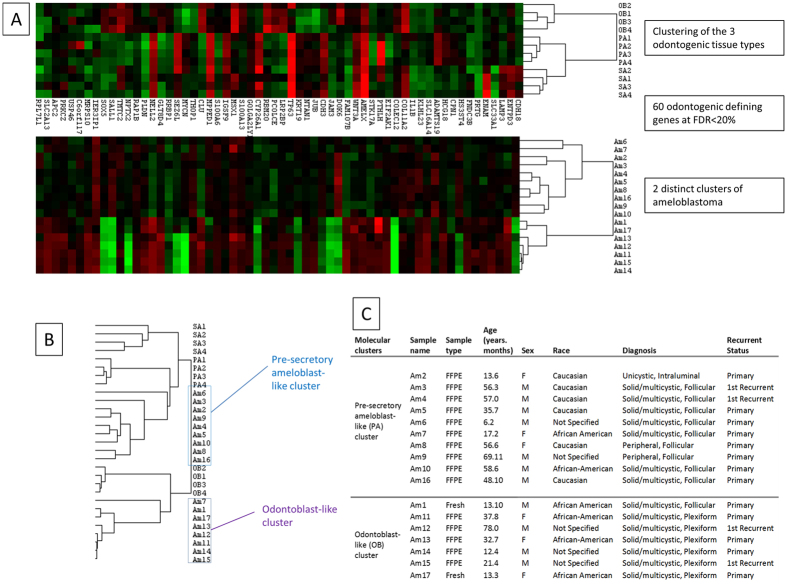
Cluster analysis used to determine reference tissue. (**A**) – Heat map of the 3 different odontogenic tissues (OB – Odontoblast, PA – Pre-secretory ameloblast, SA – Secretory ameloblast) and the 2 distinct clusters of Ameloblastoma (AM) clustered using the 60 odontogenic epithelium-defining genes. (**B**) – Array tree showing grouping of the 2 clusters with odontoblast and pre-secretory ameloblast. (**C**) – Participant demographic and tumor phenotype.

Unsupervised hierarchical cluster analysis was conducted for the tumor and normal tissue samples which showed the presence of 2 distinct clusters of ameloblastoma and a separate cluster of normal odontogenic tissue (Supplementary Fig. 2). Supervised cluster analysis using the 60 odontogenic tissue defining genes showed that the 2 clusters of ameloblastoma associated most closely with pre-secretory ameloblast (PA) and odontoblast (OB) (Fig. 2A,B). As such, these 2 clusters were designated as the pre-secretory ameloblast-like ameloblastoma (pAM) and odontoblast-like ameloblastoma (oAM), respectively. Out of 10 samples in the pAM cluster, 9 were of the follicular type while 6/7 of the samples in the oAM cluster were of the plexiform type. (Fig. 2C). A Chi-Square analysis showed that the molecular clusters were significantly associated with a histological subtype (*p* < 0.05). A single comparison tissue could not be designated as the 2 clusters associated most closely with different odontogenic tissue; instead, a multiclass approach was employed.

### Multiclass analysis

**Figure 3:**
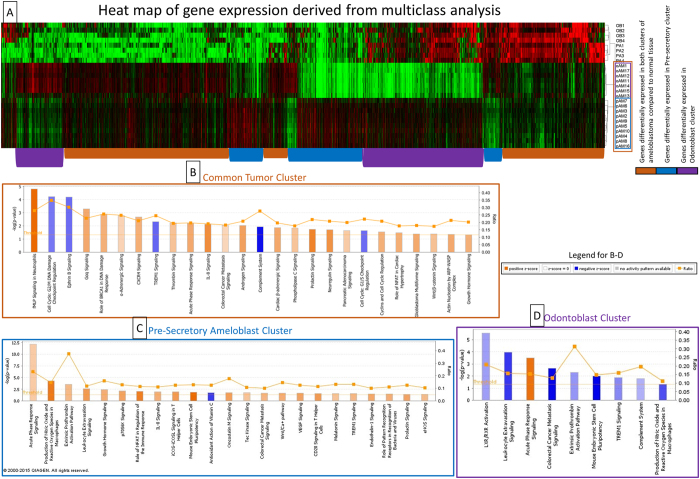
Multiclass and pathway analysis of the different tumor clusters. (**A**) – Heat map of the genes with a FDR < 1% that are differentially expressed in the 4 clusters (OB – odontoblast, PA – pre-Secretory Ameloblast, oAM – odontoblast-like ameloblastoma, pAM – pre-secretory Ameloblast-like ameloblastoma) from a SAM multiclass analysis. The cluster tree on the right shows the 2 distinct clusters of ameloblastoma. Groups of genes with similar expression (identified by colored bars at the bottom of the heat map) were used for pathway analysis for the different clusters of ameloblastoma which were shown in (**B**–**D**). (**B**) – Canonical pathways that are differentially expressed for the Common tumor cluster in IPA. (**C**) – Canonical pathways that are differentially expressed for the Pre-secretory ameloblast cluster in IPA. (**D**) – Canonical pathways that are differentially expressed for the odontoblast cluster in IPA.

Multiclass analysis was conducted between the 2 tumor clusters and 2 associated normal tissues (pAM, oAM, PA, OB) and differentially expressed genes were carried forward in a cluster analysis (Fig. 3A). To characterize the transcriptome of ameloblastoma and the 2 molecular sub-clusters, the gene expression data were analyzed in 3 groups. The common tumor cluster describes differential gene expression common in both tumor clusters compared to normal tissue and comprises 2592 genes that were expressed at a higher and lower level in the 2 tumor clusters (pAM, oAM) compared to the 2 normal tissue clusters (OB, PA). The pAM cluster describes differential gene expression unique to that cluster and consists of 1287 genes expressed at higher and lower levels compared to the other 3 groups. The oAM cluster describes differential gene expression unique to oAM tumors and consists of 1516 genes expressed at a higher and lower levels compared to the other 3 groups. The genes with fold changes at FDR < 1% were used for pathway analysis (Supplementary Table 3).

### Pathway and gene set enrichment analysis

Ingenuity pathway analysis was used to examine the activated and inhibited canonical pathways for each tumor cluster (Table 1).

The common tumor cluster had 21 activated (*z*-score > 1) and 5 inhibited (*z*-score < −1) pathways (Fig. 3B) at *p*-value < 0.05. Genes associated with notable biological processes that were differentially expressed in all the ameloblastoma tumors included prevention of damage to cell cycle regulation, cancer pathways, inflammatory pathways and Map kinase related pathways.

In addition, GSEA conducted between the common tumor cluster and normal tissues showed that 1860 out of the 2381 genes sets in the “all curated gene sets v4.0” were up-regulated in the common tumor cluster. Nineteen upregulated gene sets were significantly enriched at the nominal *p*-value < 0.05. (Supplementary Table 4).

The pre-secretory ameloblast tumor cluster had 22 activated and 1 inhibited pathway below the critical *p*-value threshold (Fig. 3C). Pathway analysis showed activation in the known cancer pathways, several inflammatory pathways and EGFR pathways.

The odontoblast tumor cluster had 1 activated and 8 inhibited pathways (Fig. 3D). Several inflammatory pathways were found to be inhibited in this cluster.

### Upstream analysis

Upstream regulators that were predicted to be activated or inhibited are listed in Table 2. Most of the predicted upstream regulators in the common tumor cluster were transcription regulators, kinases and cytokines. Specifically, several Map kinase members and inflammatory cytokines were predicted to be activated.

### Correlation with The Cancer Genome Atlas

The 2 molecular subtypes of ameloblastoma were compared with the transcriptome of the cancer subtypes in TCGA (Supplementary Fig. 3). The analysis did not show any significant correlation of ameloblastoma with any of the 13 subtypes of cancers that are well studied and has established treatment protocols. As ameloblastoma does not seem to correlate molecularly with the cancer subtypes, more investigation into ameloblastoma tumorigenesis is needed for the development of effective treatment.

## Discussion

The major finding of this study was the molecular heterogeneity of ameloblastoma that was strongly associated with its histological subtypes. Gene expression profiles of follicular and plexiform subtypes were more closely related to gene expression profiles of different normal odontogenic tissues and the follicular subtype showed activation of different molecular pathways compared with the plexiform subtype. This new knowledge can serve as a rich hypothesis-generating resource for the study of molecular and phenotypic characteristics of ameloblastoma.

One of the strengths of this study was the use of LCM for the examination of odontogenic tumors. The ability of LCM to isolate one cell-thick discrete tissue populations^23^ facilitates the targeting and pooling of neoplastic epithelial portions of ameloblastoma. Similar to the present study, Heikinheimo and colleagues found that ameloblastoma gene expression is heterogeneous, and identified 2 distinct tumor clusters with gene expression profile that were most similar to gene expression in the cap/bell stage of tooth development^12^. Using supervised cluster analysis we found that more than half of ameloblastoma samples were most similar in gene expression to pre-secretory ameloblast, similar to those observed in the early cap/bell stage as described by Heikinheimo. The remaining ameloblastoma samples were associated with the mesenchymal-derived odontoblast rather than the epithelial-derived ameloblast; this appears to be driven by differences in inflammatory pathways and was associated with a different histological appearance. However, the early pre-secretory ameloblast in this sample could be more appropriately described as preameloblast from the early cap stage of tooth development. Preameloblasts share a number of genes with odontoblasts due to significant cross-talk during differentiation; it is therefore unsurprising that a cluster of ameloblastoma was associated with odontoblast.

The examination of pathways common to both tumors shows that inflammation appears to play an important role in ameloblastoma tumorigenesis and proliferation. This finding is similar to an earlier microarray study showing increased expression levels of inflammatory mediators^13^. Canonical pathway analysis showed that several immune/inflammatory pathways are activated in addition to several predicted activated upstream cytokines. The association between dysregulated inflammation and cancer progression has been studied extensively^24^. The greater number of pathways activated in the pre-secretory cluster suggests that this process may be more important in the follicular subtype.

The Wnt signaling pathway is important in the development of ameloblastoma as discussed in the microarray study by DeVilliers^15^. Some investigators found the activation of beta-catenin downstream of Wnt signaling^25^ while others found that expression of various Wnt pathways differ among ameloblastoma with the canonical Wnt pathway being the main transduction pathway^26^. In this study, the canonical Wnt/β-catenin Signaling pathway was found to be activated in the common tumor cluster, highlighting its importance in tumorigenesis.

Additionally, both tumor clusters revealed that damage to cell cycle regulation pathways play important roles. Alteration in cell cycle regulation has been found by other investigators examining ameloblastoma gene expression^11^,^14^. A key regulator in the cell cycle damage prevention pathways is *TP53* which is also predicted to be inhibited in our upstream analysis. *TP53* is a major tumor suppression gene^27^ and the loss of a tumor suppressor gene activity in ameloblastoma may be important in the tumorigenesis process. Although the dental epithelium defining *SOX2* was not found to be differentially expressed, *SOX11* of the same family of transcription factors related to oncogenic transformation^28^ was found to be inhibited in the tumor samples.

Several canonical pathways involving the MAPK pathways and upstream members were found to be activated in the common tumor cluster. The MAPK pathways have long been considered tumor driver pathways in the pathogenesis of various cancers and are thought to be important in ameloblastoma tumorigenesis^29^,^30^. Although the expression levels of usual targets such as *BRAF* and *EGFR* were not directly increased, activation of the pathway suggests alterations in the activity of the members. Recently, Kurppa and colleagues found *BRAF* gene mutations, specifically V600E mutation, in 63% of ameloblastomas^31^. *BRAF* is in the RAS pathway and MAPK cascade. This mutation leads to a 500 fold increase in activity of *BRAF* and increases signals through *MEK* to activate *ERK*^32^. *ERK* is the activator of numerous downstream transcription factors which induces biochemical functions such as cell differentiation, proliferation, growth, and apoptosis. The V600E mutation in *BRAF* is a promising oncogene target for the anti-neoplastic drug dabrafenib; which was used in conjunction with a *MEK* inhibitor in a patient with stage 4 ameloblastoma with good results^33^. The use of chemotherapeutic agents to reduce tumor size can be very helpful in cases of ameloblastoma requiring extensive surgical margins and major post-surgical reconstruction^34^.

In GSEA, the gene sets with the highest activation scores, were cancer related including “SMID_BREAST_CANCER_LUMINAL_A_DN”. Cancer related pathways were also found to be differentially expressed in our pathway analysis. Moreover, breast cancer specific “Role of BRCA1 in DNA Damage Response” pathway was found to be differentially expressed, with the 2 different analyses highlighting similar molecular pathways.

One of the short-comings of this study is that most of the samples were FFPE. Formalin fixing can cause the degradation of RNA^35^ and affect the accuracy of microarrays. However, recent studies supported the use of such samples for gene expression analysis^36^ and NanoString has been shown to produce consistent results independent of the sample type (fresh frozen versus FFPE)^37^. Genes in our microarray data that had the greatest fold changes showed good correlation with the nanoString expression. In addition, there were no outliers in the cluster analysis among the ameloblastoma cluster analysis indicating consistent results between fresh frozen and FFPE samples.

Strengths of the study included the use of LCM and universal RNA as a means of normalizing between arrays. Ameloblastoma presents with neoplastic epithelial tissue surrounded by stromal tissue making isolation very difficult. As a result, most investigations of ameloblastoma used samples that contain diverse cell populations such as the surrounding stroma which can obscure driver pathways from the actual neoplastic epithelial cells. The use of the universal RNA facilitated the use of the dentome as a comparison and also allows the use of the microarray data by other investigators using universal RNA for normalization.

In conclusion, our study isolated ameloblastoma epithelial and normal odontogenic cells using LCM to identify gene expression profiles and molecular pathways that are potentially important in the tumorigenesis of ameloblastoma. Ameloblastoma showed 2 distinct molecular profiles that were associated with different histological subtypes suggesting they could be receptive to different chemotherapeutic protocols. These results provide a wealth of information that can be used in future experimental and mechanistic studies, involving animal models and new pharmacogenomic approaches.

## Additional Information

**How to cite this article**: Hu, S. *et al.* Ameloblastoma Phenotypes Reflected in Distinct Transcriptome Profiles. *Sci. Rep.* **6**, 30867; doi: 10.1038/srep30867 (2016).

## Supplementary Material

Supplementary Information

## Figures and Tables

**Table 1:** Canonical pathways differentially expressed in ameloblastoma clusters.

Canonical pathways different between the ameloblastoma common tumor cluster and normal cells				
Biological process	Ingenuity Canonical Pathways	−log(p-value*)	Ratio	z-score
Inflammatory (Immune) response/cytokine signaling	fMLP Signaling in Neutrophils	4.81	0.28	3.674
	CXCR4 Signaling	2.68	0.21	1.961
	TREM1 Signaling	2.34	0.25	−1.213
	Thrombin Signaling	2.21	0.19	1.512
	Acute Phase Response Signaling	2.20	0.20	2.117
	IL-8 Signaling	2.12	0.19	2.785
	Complement System	1.94	0.28	−1.89
Cell cycle regulation	Cell Cycle: G2/M DNA Damage Checkpoint Regulation	4.23	0.35	−1.069
	Role of BRCA1 in DNA Damage Response	2.88	0.26	1.941
	Cell Cycle: G1/S Checkpoint Regulation	1.63	0.22	−1.155
	Cyclins and Cell Cycle Regulation	1.53	0.21	1.941
Cancer	Colorectal Cancer Metastasis Signaling	2.04	0.18	1
	Pancreatic Adenocarcinoma Signaling	1.63	0.20	1.291
	Glioblastoma Multiforme Signaling	1.40	0.18	2.132
	Wnt/β-catenin Signaling	1.37	0.18	1.528
	Actin Nucleation by ARP-WASP Complex	1.35	0.21	2.111
Map kinase related	Gαq Signaling	3.31	0.23	2.117
	α-Adrenergic Signaling	2.77	0.25	1.069
	Phospholipase C Signaling	1.83	0.18	1.461
	Prolactin Signaling	1.74	0.22	3.051
Receptor tyrosine kinase (RTK)	Ephrin B Signaling	4.21	0.30	−1.155
	Neuregulin Signaling	1.69	0.21	2.668
Nuclear receptor signaling	Androgen Signaling	2.01	0.21	2.333
Cellular growth and proliferation	Growth Hormone Signaling	1.32	0.20	1.941
Others	Cardiac β-adrenergic Signaling	1.87	0.20	2.138
	Role of NFAT in Cardiac Hypertrophy	1.47	0.18	1.961
**Canonical pathways different for the ameloblastoma presecretory cluster**				
Inflammatory (Immune) response/cytokine signaling	Acute Phase Response Signaling	12.20	0.23	1.225
	Production of Nitric Oxide and Reactive Oxygen Species in Macrophages	4.26	0.15	3.138
	Extrinsic Prothrombin Activation Pathway	3.55	0.38	1.633
	Leukocyte Extravasation Signaling	2.63	0.12	2.324
	Role of NFAT in Regulation of the Immune Response	2.08	0.11	3
	IL-8 Signaling	1.95	0.11	1.886
	iCOS-iCOSL Signaling in T Helper Cells	1.91	0.13	2.333
	Antioxidant Action of Vitamin C	1.80	0.13	−1.897
	CD28 Signaling in T Helper Cells	1.60	0.12	2.333
	TREM1 Signaling	1.55	0.13	1.667
	Role of Pattern Recognition Receptors in Recognition of Bacteria and Viruses	1.44	0.11	1.897
	eNOS Signaling	1.35	0.10	1.604
Cellular growth and proliferation	Growth Hormone Signaling	2.44	0.16	1.897
	p70S6K Signaling	2.15	0.13	2.138
	Mouse Embryonic Stem Cell Pluripotency	1.84	0.13	3.464
	Oncostatin M Signaling	1.75	0.18	2.236
	VEGF Signaling	1.63	0.12	2.121
Protein tyrosine kinase (PTK)	Tec Kinase Signaling	1.73	0.11	1.387
Cancer	Colorectal Cancer Metastasis Signaling	1.71	0.10	1.706
	Wnt/Ca+ pathway	1.68	0.15	1.89
Others	Melatonin Signaling	1.59	0.13	1.414
	Endothelin-1 Signaling	1.45	0.10	2
	Prolactin Signaling	1.41	0.12	2.121
**Canonical pathways different for the ameloblastoma odontoblast cluster**				
Inflammatory (Immune) response/cytokine signaling	Leukocyte Extravasation Signaling	3.99	0.16	−2.524
	Acute Phase Response Signaling	3.51	0.16	1.897
	Extrinsic Prothrombin Activation Pathway	2.30	0.31	−1.342
	TREM1 Signaling	1.89	0.16	−1.508
	Complement System	1.80	0.19	−1
	Production of Nitric Oxide and Reactive Oxygen Species in Macrophages	1.33	0.11	−2.236
Nuclear receptor signaling	LXR/RXR Activation	5.57	0.21	−1.225
Cancer	Colorectal Cancer Metastasis Signaling	2.67	0.13	−3.157
Cellular growth and proliferation	Mouse Embryonic Stem Cell Pluripotency	2.01	0.15	−3.207

*p-value  <  0.05.

**Table 2:** Upstream analysis using Ingenuity Pathway Analysis.

Activated and inhibited molecules in Common Tumor Cluster				
Molecule Type	Upstream Regulator	Predicted Activation State	Activation z-score	p-value of overlap
transcription regulator	SOX11	Inhibited	−2.16	0.47
	NUPR1	Inhibited	−2.46	0.00
	NEUROG1	Inhibited	−2.18	0.00
	KDM5B	Inhibited	−3.66	0.00
	TP53	Inhibited	−2.37	0.00
	HIF1A	Activated	2.90	0.00
	JUN	Activated	2.55	0.00
	FOXM1	Activated	2.99	0.00
	EZH2	Activated	2.06	0.10
	YAP1	Activated	2.21	0.01
	MYB	Activated	2.39	0.00
kinase	TRIB3	Inhibited	−2.00	0.10
	CDKN1A	Inhibited	−2.60	0.00
	AKT1	Activated	2.65	0.01
	EGFR	Activated	2.20	0.04
	MAPK9	Activated	2.31	0.02
	PIM1	Activated	2.42	0.05
	MAP3K14	Activated	2.21	0.13
	EIF2AK2	Activated	2.43	0.01
	ERBB2	Activated	2.46	0.00
cytokine	CSF2	Activated	2.43	0.01
	TNF	Activated	2.27	0.00
	IL1A	Activated	2.12	0.00
	IL6	Activated	3.33	0.01
	OSM	Activated	2.41	0.00
enzyme	STUB1	Inhibited	−2.24	0.03
	HRAS	Activated	2.16	0.00
ligand-dependent nuclear receptor	PGR	Activated	3.22	0.00
	ESRRA	Activated	2.20	0.07
transporter	SLC29A1	Activated	2.00	0.14
	S100A6	Activated	2.12	0.01
complex	IgG	Inhibited	−2.04	0.00
	NFkB (complex)	Activated	2.35	0.00
	Cg	Activated	2.69	0.00
group	STAT5a/b	Activated	2.00	0.55
	Mek	Activated	2.36	0.05
	Growth hormone	Activated	2.55	0.06
transmembrane receptor	TREM1	Activated	2.82	0.00
growth factor	NRG1	Activated	2.17	0.06
other	RBM5	Inhibited	−2.34	0.07
	UXT	Inhibited	−2.44	0.01
	AHI1	Activated	2.00	0.24
	RABL6	Activated	3.30	0.00
	HSPB2	Activated	2.22	0.00
**Activated and inhibited molecules in Presecretory Cluster**				
enzyme	MGEA5	Inhibited	−2.04	0.00
ligand-dependent nuclear receptor	PPARG	Activated	2.01	0.00
transcription regulator	NUPR1	Activated	2.11	0.00
growth factor	WISP2	Activated	2.24	0.06
**Activated and inhibited molecules in Odontoblast Cluster**				
transcription regulator	SOX11	Inhibited	−2.98	0.05
	MDM2	Inhibited	−2.24	0.04
	CEBPA	Inhibited	−2.14	0.00
	NUPR1	Inhibited	−2.47	0.36
	TP53	Inhibited	−2.19	0.01
enzyme	TGM2	Inhibited	−3.59	0.00
	HMOX1	Activated	2.00	0.27
ligand-dependent nuclear receptor	AR	Inhibited	−2.24	0.25
	NR3C1	Inhibited	−2.56	0.17
transporter	S100A6	Activated	2.45	0.01
group	estrogen receptor	Inhibited	−2.28	0.00
