# Supplementary material for: Ameloblastoma Phenotypes Reflected in Distinct Transcriptome Profiles
Source: Sci Rep. 2016 Aug 5;6:30867. doi: 10.1038/srep30867 (PMC4974613; doi:10.1038/srep30867)
Supplement: Supplementary Information [file srep30867-s1.pdf]

Supplementary Information

Ameloblastoma Phenotypes Reflected in Distinct Transcriptome Profiles

Shijia Hu[1,2], Joel Parker[3], Kimon Divaris[1,4], Ricardo Padilla[5], Valerie Murrah[5], John Timothy Wright[1]

[1]Pediatric Dentistry, School of Dentistry University of North Carolina-Chapel Hill, Chapel Hill, NC, USA

[2]Faculty of Dentistry, National University of Singapore, Singapore

[3]Cancer Genetics, University of North Carolina-Chapel Hill, Chapel Hill, NC, USA

[4]Epidemiology, Gillings School of Global Public Health, University of North Carolina-Chapel Hill, Chapel Hill, NC, USA

[5]Diagnostic Sciences, School of Dentistry, University of North Carolina-Chapel Hill, Chapel Hill, NC, USA

Supplementary Figure 1 – Scatterplot of Microarray (x-axis) differential expression versus NanoString (y-axis) differential expression.

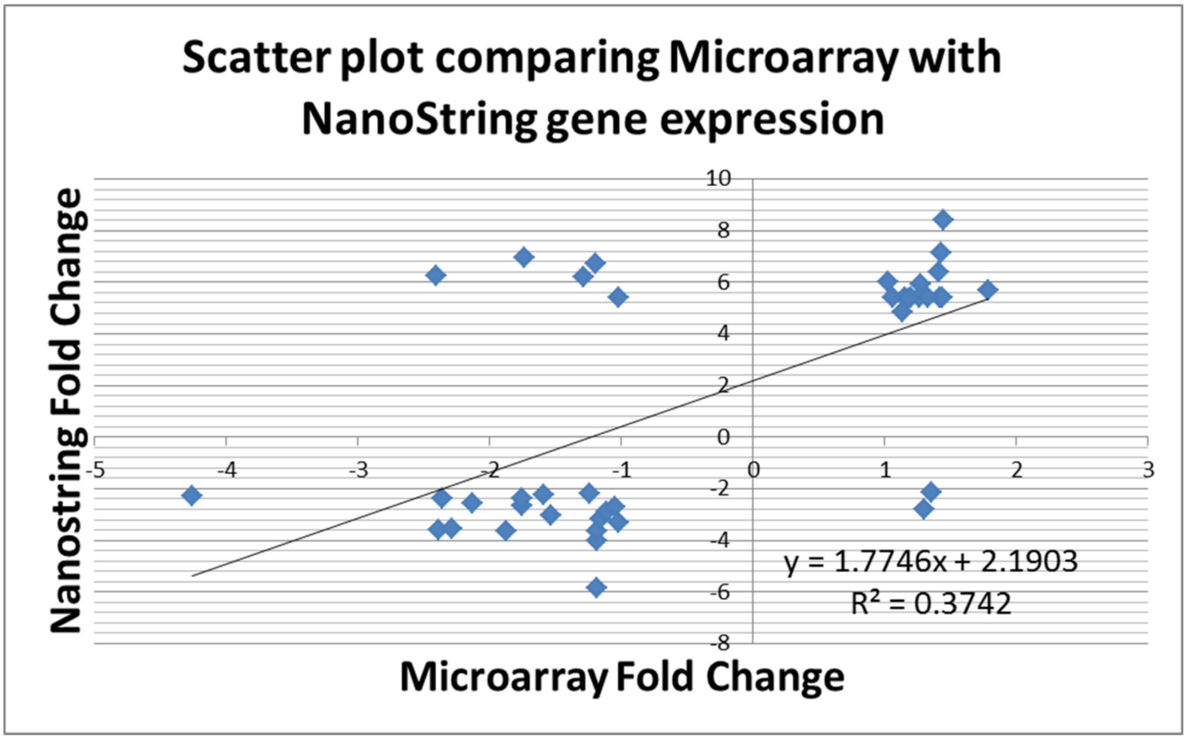

Supplementary Figure 2 – Unsupervised hierarchical cluster analysis of reference tissue with tumor samples. 3126 genes with the greatest standard deviation between the samples were used for the cluster analysis.

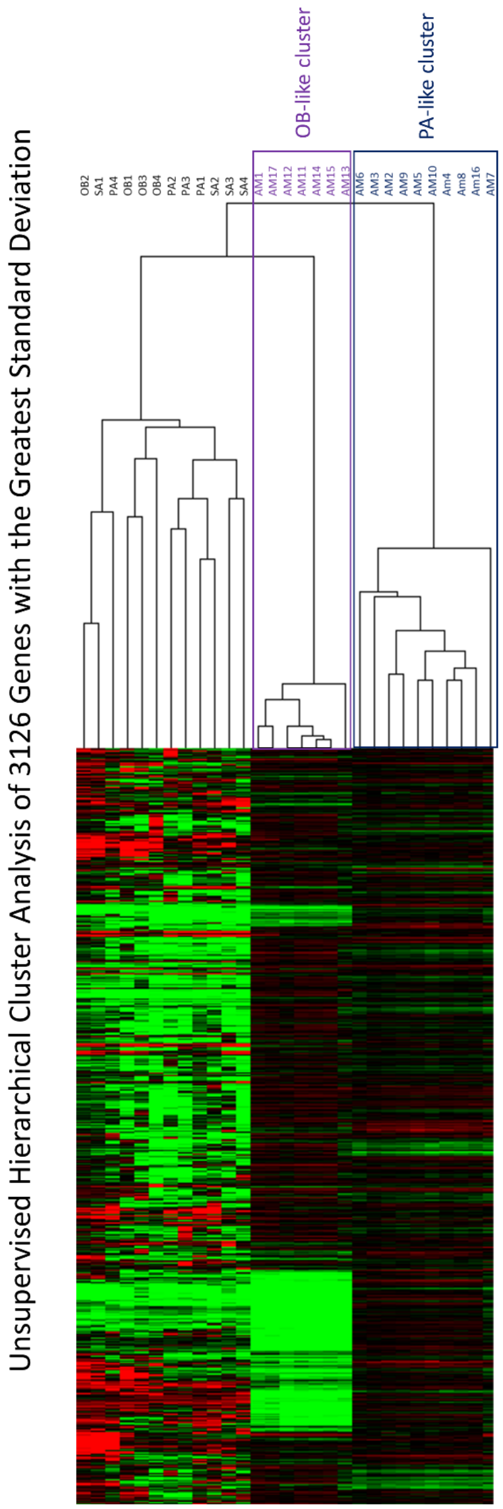

Supplementary Figure 3 – Correlation analysis with The Cancer Genome Atlas subtypes suggesting no significant correlation between ameloblastoma and the 13 cancer subtypes.

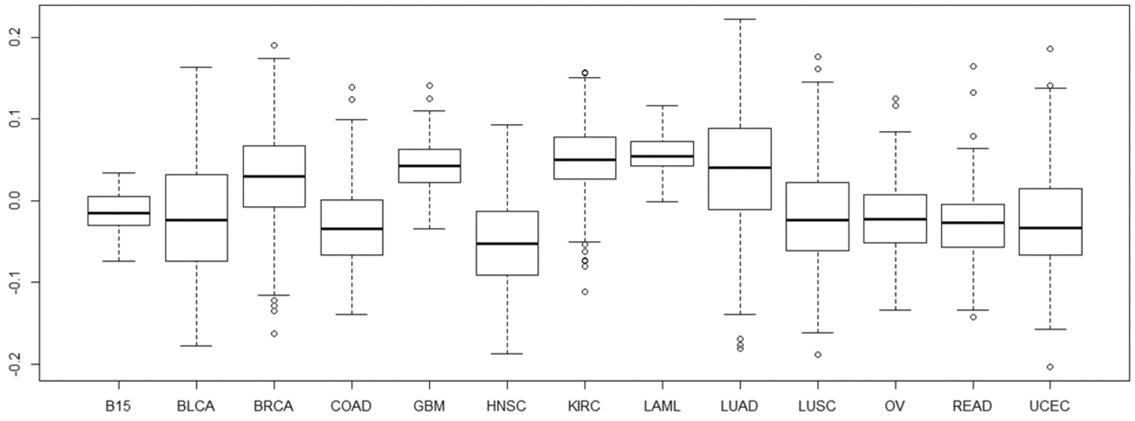

Supplemental Table 1

Tumor samples RNA yield and quality					
**Sample Name**	**Tissue type**	**Nanodrop assessment**			
		**ng/µl**	**A 260/280**	**A 260/230**	**yield (ng)**
Am1	Fresh	13.1	2.45	1.64	195.9
Am2	FFPE	4.4	1.7	0.4	88.0
Am3	FFPE	46.4	2.1	1.5	928.0
Am4	FFPE	41.3	2.0	0.7	826.0
Am5	FFPE	35.5	1.8	0.6	710.0
Am6	FFPE	7.6	1.6	0.6	152.0
Am7	FFPE	19.2	1.9	1.0	384.0
Am8	FFPE	15.9	2.1	1.1	318.0
Am9	FFPE	27.2	2.1	1.4	544.0
Am10	FFPE	23.5	1.9	0.5	470.0
Am11	FFPE	46.9	2.15	2.00	703.8
Am12	FFPE	15.5	1.43	0.08	309.8
Am13	FFPE	8.6	2.04	0.86	172.4
Am14	FFPE	5.1	1.85	0.30	101.1
Am15	FFPE	21.4	1.74	0.18	427.2
Am16	FFPE	10.2	1.7	1.2	204.0
Am17	Fresh	5.8	2.55	1.17	87.2

Supplementary Table 2

Top 20 upregulated genes in ameloblastoma compared to pre-secretory ameloblast

Gene Name	NanoString Fold Change	Microarray Fold change
IL1A	8.39	1.45
IL8	7.16	1.43
AREG	6.96	-1.74
IL1B	6.74	-1.19
MMP1	6.38	1.41
SERPINE1	6.23	-2.41
CASP10	6.19	-1.29
CSF3	6.03	1.02
MMP3	5.91	1.27
NTRK1	5.67	1.79
CYP1A1	5.43	1.41
GATA1	5.43	1.26
IL4	5.43	1.06
MPL	5.43	1.15
PLG	5.43	1.44
TERT	5.43	-1.02
WEE1	5.43	1.20
RET	5.40	1.33
IL6	5.36	1.18
EGF	4.84	1.13

Top 20 downregulated genes in ameloblastoma compared to pre-secretory ameloblast

Gene Name	NanoString Fold Change	Microarray Fold change
SPP1	-5.83	-1.19
TYMS	-4.03	-1.18
KDR	-3.67	-1.18
DLC1	-3.63	-1.87
KRAS	-3.59	-2.39
MYC	-3.53	-2.29
FYN	-3.29	-1.02
MSH6	-3.17	-1.16
TOP2A	-3.05	-1.53
CCNA2	-2.91	-1.12
COL1A1	-2.79	1.30
JUNB	-2.73	-1.05
IGF1	-2.68	-1.76
PDGFRA	-2.56	-2.13
EPS8	-2.36	-2.36
CCND2	-2.36	-1.76

CAV1	-2.30	-4.27
CDKN2C	-2.24	-1.59
TGFBR3	-2.21	-1.24
FOS	-2.12	1.36

Supplementary Table 3 – Genes in each cluster used for pathway analysis FDR < 1%

Data uploaded at
http://genomewide.net/public/transcriptome/ameloblastoma/Supplemental_Table_2.xlsx

Supplementary Table 4

Upregulated gene sets of the ameloblastoma common tumor cluster compared to normal cells				
**Gene Set**	**Number of genes**	**Enrichment Score**	**Normalized enrichment score**	**Nominal p-value**
GAUSSMANN_MLL_AF4_FUSION_TARGETS_F_DN	12	0.70	1.57	> 0.01
LEE_LIVER_CANCER_E2F1_DN	14	0.58	1.68	> 0.01
BROWNE_HCMV_INFECTION_10HR_UP	24	0.45	1.55	> 0.01
PACHER_TARGETS_OF_IGF1_AND_IGF2_UP	14	0.54	1.52	0.01
TONKS_TARGETS_OF_RUNX1_RUNX1T1_FUSION_SUSTAINDED_IN_ERYTHROCYTE_UP	14	0.61	1.68	0.01
HOUSTIS_ROS	15	0.63	1.47	0.01
NAKAYAMA_SOFT_TISSUE_TUMORS_PCA1_UP	22	0.52	1.72	0.02
SINGH_NFE2L2_TARGETS	10	0.77	1.43	0.02
GERHOLD_ADIPOGENESIS_UP	19	0.49	1.62	0.02
SENESE_HDAC1_AND_HDAC2_TARGETS_UP	76	0.42	1.31	0.03
DACOSTA_UV_RESPONSE_VIA_ERCC3_COMMON_UP	20	0.56	1.40	0.03
KEGG_METABOLISM_OF_XENOBIOTICS_BY_CYTOCHROME_P450	12	0.56	1.59	0.03
LEE_LIVER_CANCER_CIPROFIBRATE_DN	12	0.47	1.52	0.03
KRIGE_AMINO_ACID_DEPRIVATION	13	0.65	1.39	0.03
MANTOVANI_VIRAL_GPCR_SIGNALING_UP	28	0.37	1.42	0.03
SU_LIVER	14	0.54	1.63	0.04
ADDYA_ERYTHROID_DIFFERENTIATION_BY_HEMIN	21	0.54	1.42	0.04
KERLEY_RESPONSE_TO_CISPLATIN_UP	11	0.51	1.57	0.04
SMID_BREAST_CANCER_LUMINAL_A_DN	12	0.62	1.45	0.05
